# Music Attenuates Excessive Visual Guidance of Skilled Reaching in Advanced but Not Mild Parkinson's Disease

**DOI:** 10.1371/journal.pone.0006841

**Published:** 2009-08-31

**Authors:** Lori-Ann R. Sacrey, Callie A. M. Clark, Ian Q. Whishaw

**Affiliations:** Canadian Centre for Behavioural Neuroscience, University of Lethbridge, Lethbridge, Alberta; Hotchkiss Brain Institute, University of Calgary, Canada

## Abstract

Parkinson's disease (PD) results in movement and sensory impairments that can be reduced by familiar music. At present, it is unclear whether the beneficial effects of music are limited to lessening the bradykinesia of whole body movement or whether beneficial effects also extend to skilled movements of PD subjects. This question was addressed in the present study in which control and PD subjects were given a skilled reaching task that was performed with and without accompanying preferred musical pieces. Eye movements and limb use were monitored with biomechanical measures and limb movements were additionally assessed using a previously described movement element scoring system. Preferred musical pieces did not lessen limb and hand movement impairments as assessed with either the biomechanical measures or movement element scoring. Nevertheless, the PD patients with more severe motor symptoms as assessed by Hoehn and Yahr (HY) scores displayed enhanced visual engagement of the target and this impairment was reduced during trials performed in association with accompanying preferred musical pieces. The results are discussed in relation to the idea that preferred musical pieces, although not generally beneficial in lessening skilled reaching impairments, may normalize the balance between visual and proprioceptive guidance of skilled reaching.

## Introduction

Parkinson's disease (PD) is characterized by motor, sensory, and attentional impairments [1]–[2], and is related to a progressive degeneration of dopamine producing neurons in the substantia nigra pars compacta [3]. Motor symptoms are manifest in many laboratory-based motor tasks [4]–[5] and analogues of real-world tasks [6]–[8]. Impairments in movement are often accompanied by impairments in sensation [9]–[12]. For example, studies on walking suggest that PD subjects are more dependent on visual guidance than are control subjects [9]–[10] and studies on memory-guided pointing demonstrate that PD subjects are less accurate than control subjects in the absence of vision [11]–[12]. It should therefore not be surprising that movement impairment has been shown to improve under sensory cueing [13]–[16]. For example, when pointing to remembered targets, PD subjects make errors, but when given an external visual cue to point at, errors are reduced [17]. Similarly, providing PD subjects with verbal instructions (i.e., take long steps) versus self-selected gait patterns [14], [18], or placing lines on the floor to serve as sensory cues [19] can improve cadence, stride length, and velocity of gait. Consequently, it is not always clear the extent to which a given impairment relates to motor, sensory, or attentional deficits.

A number of lines of research have examined the effects of music as a sensory cue to assist in overcoming PD deficits [1], [15], [20]–[22]. Music is shown to lessen whole body bradykinesia to the point that otherwise immobile PD patients can dance and it has also be reported to improve utensil usage [20]. These results suggest a very general beneficial effect of music on whole body movement and skilled movements. At present, it is unclear what aspects of skilled movement are improved under the effects of music. One form of skilled movement, the reach-to-eat task, in which a subject reaches for a small food item, grasps it, and transports it to the mouth for eating, provides a sensitive assay of PD effects on skilled limb movement [23]–[25]. PD subjects are slow to complete the movement [8], are impaired in rotatory movements of the limb, and are impaired in shaping the hand to grasp [23], [26]. The impairments persist with medication [24]. The fact that limb movements in reaching are sensitive to the effects of PD and are resistant to improvement with drug medication provides an opportunity for evaluating whether accompanying preferred musical pieces lessens the impairments in sensory and motor control.

In the present study, young adult control subjects, age-matched control subjects, and adults with mild and advanced PD were instructed to reach for and eat a small food item. Subjects were fitted with light reflective markers to measure arm and hand movement, wore an eye–tracking system to monitor eye movements, and were fitted with goggles that could be manipulated to occlude visual feedback during the reach. On some trials, subjects listened to preferred musical pieces. Synchronized data from the light reflective markers and the eye-tracking system were compiled to determine the contribution of visual guidance to skilled reaching, and the effects of preferred musical pieces on skilled reaching, movement element scoring, and visual guidance of skilled reaching.

## Materials and Methods

### Subjects

On the basis of Hoehn and Yahr (HY) scores [27], PD subjects were divided into two groups, mild PD (HY<2.5; 6 females and 2 males; ages 63.88±8.32 years; HY = 1.93±0.56) and advanced PD (HY>2.5; 3 females and 4 males; ages 75.00±6.68 years; HY = 3.07±0.67). Because skilled reaching is not affected by medication [24], the subjects were ON regular medication at the time of testing. For PD subject characteristics, see Table 1. Age-matched old adult control (OAC) subjects were recruited from the city of Lethbridge (8 females and 7 males; ages 62.80±7.52 to 81.71±5.02 years). Eleven young adult control (YAC) subjects (4 females and 7 males; ages 22.27±3.85 years) were recruited from the University of Lethbridge campus. All control subjects were self-reported to be of good health with no history of neurological disorder, and had normal or corrected to normal (contact lens) vision.

**Table 1:** Parkinson's diseased subjects' characteristics.

Subject ID	Group	Age	Sex	Hoehn and Yahr	Medications
1	Advanced	64	Male	2.5	Sinemet; Amantadine
2	Advanced	71	Male	2.5	Sinemet
3	Mild	61	Female	2	Mirapex
4	Mild	75	Male	2	Sinemet
5	Mild	70	Female	1.5	Levodopa; Ropinirole
6	Mild	72	Female	2	Sinemet
7	Mild	57	Female	2	Sinemet; Ropinirole
8	Mild	61	Female	2	Sinemet; Amantidine
9	Mild	50	Female	1	Carbidopa; Mirapex; Amantidine
10	Advanced	74	Male	2.5	Sinemet
11	Mild	65	Male	2	Sinemet
12	Advanced	75	Female	4	Sinemet
13	Advanced	84	Female	4	Sinemet
15	Advanced	67	Male	3	Sinemet

### Ethics Statement

The University of Lethbridge Human Subject Research Committee approved the study. Rationale for the experiments and testing information were listed on a written consent form that each subject was required to read and sign prior to initiation of testing. The study was conducted in accordance with the Declaration of Helsinki.

### Reaching Task

**Figure 1:**
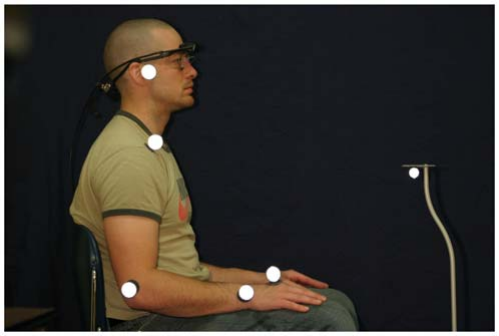
Experimental set-up. The white dots represent light reflective markers on the subject (left) and the food target (right). The head set is for eye-tracking. Food is placed on the pedestal and the subject begins the first reach with hand open on the lap.

Subjects performed a seated skilled reaching task in which they reached toward the top of a pedestal for a small food item that was grasped and transported to the mouth for eating [23]–[25]. Subjects were seated in a comfortable upright position, with their feet flat on the floor (Figure 1). The self-standing height adjustable pedestal was placed directly in front of the subject at a horizontal reach amplitude normalized to the subjects' arm length (100% of length from shoulder to tip of index finger with elbow at 180° flexion) and a vertical amplitude normalized to the subjects' trunk height (100% of height from floor to outstretched arm while seated and with shoulder at 90° flexion).

### Reaching instructions

Once subjects were seated, they were asked to place their hands palm down on their thighs, and this instruction was not repeated. The experimenter stood to the left of the subject (i.e. in peripheral visual space) and placed a food item (Cheerio™) on the pedestal for each trial. The subjects were instructed to reach for food with their dominant (right) hand. Each testing trial was initiated with a verbal “ready” signal, immediately followed by a verbal “go” signal as a permissive cue to the subject to start the trial at their leisure. Each trial concluded following successful placement of the food item in the mouth and return of the reaching hand to its start position on the lap. The experimenter maintained a casual relationship with the subjects, i.e., engaging in conversation, in order to maintain a quasi-natural testing condition. Because subjects were not informed their eye movements were under investigation, they were given no instructions concerning where they were to look during testing.

### Reach measurement

Skilled reaching was measured using biomechanical markers [25], eye-tracking glasses [25], and movement element scoring [23]–[24].

#### (1) Biomechanical measurement of reaching

Subjects were fitted unilaterally (right side) with reflective markers at (a) zygomatic bone, (b) acromion process, (c) lateral epicondyle of the humerus, and (d) ulnar styloid process. A reflective marker was also placed on the pedestal under the target platform. A digital video camera was positioned sagittal to the subject to record a reach-side view of the subject from lower leg to head at a sampling frequency of 500 frames per second (f/s). Trial reaches were digitized using the Peak Motus v. 8.3.0 2-D digitizing system (Peak Performance Technologies, Inc., Centennial, CO) to digitize the reflective markers on the image. Marker data were filtered using a Butterworth low-pass filter. Velocity data of the ulnar styloid process (reach wrist) were subsequently calculated (Peak Motus). The events of movement onset and offset were determined from the resultant reach wrist velocity using a custom-written algorithm (Microsoft Excel 2002), with minimal resultant velocity used to indicate the onset and offset events for the movement phases inherent to skilled reaching. Specifically, the reach-to-grasp phase (hereafter referred to as *advance*) was defined as the time between initial velocity onset (i.e. first movement of the hand) and the subsequent point of minimal velocity (i.e. as the hand contacts the food item). The grasp-to-eat phase (hereafter referred to as *withdrawal*) was defined as the time between the second velocity onset (i.e. first movement of hand away from pedestal) and the subsequent point of minimal velocity (i.e. as the food item contacts the mouth). *The total reach duration* was defined as the time between initial velocity onset (i.e. first movement of the hand) and the second subsequent point of minimal velocity (i.e. as the food item contacts the mouth).

#### (2) Biomechanical measurement of eye tracking

Subjects wore a head-mounted infrared eye tracking system (MobileEye v. 1.2, Applied Science Laboratories, Bedford, MA) to track eye movements with a sampling frequency of 60 Hz [25]. The MobileEye system uses Dark Pupil Tracking to compute the x and y coordinates of the pupil within the scene. In this technique, a set of three harmless near infrared lights are projected onto the eye, and reflected by the cornea (corneal reflection). By comparing the relative vectors from the sensor to the pupil and the cornea, the eye tracking system computes the position of the eye (point of gaze) relative to the scene. The video record of the data collected by the eye tracking system were subjected to off-line analysis to determine the following events of visual guidance: engage to move, grasp to disengage, and total engagement period. *Engage to move* was defined as the time between the first point that the eyes descend to visually fixate the food item and first movement of the forelimb towards the food item, and *grasp to disengage* was defined as the time between contact of the food item with the digits and the first point that the eyes ascend from the food item. *The total visual engagement period* was defined as the time between the first point that the eyes descend to fixate the food item (*engage*) and the first point that the eyes ascend (*disengage*) from the food item. A visual marker presented at the onset of the testing session was used to time-synchronize the video record of the biomechanical markers from the digital camera and the video record from the eye-tracking system offline using Final Cut Pro HD v.4.5 for Mac OS X v.10.2.8.

#### (3) Movement element scoring

One reach trial performed with vision for each subject was scored according to the skilled reaching scale [23] to confirm that the sample population in the present study is representative of healthy and PD populations. One reach trial performed with occlusion (see below) was also scored for each subject to compare the effect of occlusion on skilled reaching, and one reach trial performed with accompanying preferred musical pieces (see below) was scored to compare the effects of music on skilled reaching. The scored reaches were the first test reach of the vision, occlusion, and music conditions, respectively, as per methodology used in previous papers [23]–[24]. The scale is an extension of a traditional method of movement analysis [28], consisting of 21 items combined into eight temporally sequenced elements. For each of the eight elements, a score of 0 was given if the movement was present and normal, 0.5 if the item was present but abnormal, and a score of 1 was given if the movement was absent [for a full description], [ see 23–24].

### Visual occlusion

Subjects were fitted with PLATO vision-occluding goggles (Translucent Technologies, Toronto, ON) which were manipulated to allow vision (i.e., transparent) or occlude vision (i.e., opaque) [8], [25]. The goggles were modified to occlude both central and peripheral vision by attachment of a peripheral vision blocker (i.e. black felt around the perimeter and fastened to the face with porous tape). Prior to the initiation of each trial, the occlusion goggles were either opened by the experimenter for a vision trial or remained closed for an occluded trial.

### Preferred musical pieces

Prior to initiation of the testing session, subjects were asked to select two songs from their favorite artist. The self-selected music was played on a personal listening device (iPod, Apple, Cupertino, CA) during reaching in the music condition. Volume was adjusted to a comfortable level by each subject. The music was not embedded with rhythmic auditory stimulation.

### Procedure

#### Experiment 1: Skilled reaching with eye tracking

Fourteen PD (eight mild, six advanced), fifteen old adult control and eleven young adult control subjects were given the opportunity to reach for a maximum of five practice trials of the reaching task. Following the practice trials, subjects completed ten trials of the reaching task.

#### Experiment 2: Skilled reaching with visual occlusion

Thirteen PD (eight mild, five advanced), fifteen old adult control and eleven young adult control subjects were given the opportunity to reach for a maximum of four practice trials of the reaching task with vision, and a maximum of four practice trials without vision. Following the practice trials, subjects completed: (1) ten trials with vision, and (2) ten trials without vision. The 20 test trials were randomized for each subject with vision and occlusion trials intermixed using the randomizing software of Microsoft Excel.

#### Experiment 3: Skilled reaching with preferred musical pieces

Fourteen PD (eight mild, six advanced), fifteen old adult controls, and eleven young adult control subjects were given the opportunity to reach for a maximum of five practice trials of the reaching task without music. Following the practice trials, subjects completed ten trials of the reaching task without music followed by ten trials of the reaching task with preferred music. This procedure was chosen to avoid any potential carry-over effects of preferred musical pieces on non-music reaches.

### Statistical Analysis

Data were analyzed using repeated measures ANOVA (Statistical Package for the Social Sciences, SPSS v. 13). Because there were no statistical differences between the age-matched control groups for the mild PD and advanced PD groups, they were collapsed into a single group (OAC). For each subject, mean values for the test trials were calculated for each dependent variable in each condition. Bonferroni correction for post-hoc tests was used for all pairwise comparisons. Paired samples *t*-tests were performed on each group (YAC, OAC, mild PD, advanced PD) to compare the kinematics of reaches with vision to those with occlusion. We restricted comparisons between vision and occlusion and no accompanying music to accompanying music performance to those PD subjects who completed trials in both conditions

## Results

### Experiment 1: Skilled reaching with eye-tracking

All subjects performed reach trials successfully (i.e. grasped the food item and placed it in the mouth successfully on each trial). The biomechanical measurements of reaching indicated that the reaching movement slowed with age and also as a function of PD, thus advanced PD subjects reached more slowly than the YAC, OAC, and mild PD subjects. The biomechanical measurements of eye tracking indicated that all of the groups, with the exception of the advanced PD group, visually fixated the food item immediately prior to initiating arm movement towards the food item and then disengaged the food item just as they grasped it with their digits. Thus, the relationship between visual engagement of the target and the transport phase of the reaching was extremely close. This relationship was not observed in the PD subjects of the advanced PD group. Rather, they visually fixated the food item for an extended period prior to initiating arm movement towards the food item and remained fixated on the food item as it was transported to the mouth. The movement elements scoring indicated that both the mild and advanced PD groups displayed movement element impairments relative to the control groups. These main findings were confirmed statistically as is described fully below:

#### (1) Biomechanical measurement of reaching

**Figure 2:**
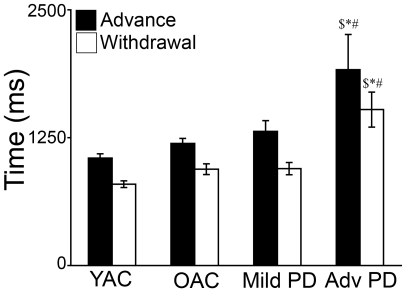
Time (mean and standard error) to complete advance and withdrawal for the four experimental groups. ▒ = advanced PD>YAC; * = advanced PD>OAC; ## = advanced PD>mild PD and # ps<0.001.

The results of the biomechanical measurements of reaching are summarized in Figure 2. A 4×2 ANOVA was performed on the movement time using GROUP (YAC, OAC, Mild PD, Advanced PD) and PHASE (advance, withdrawal) as independent variables. There was a significant effect of GROUP (*F*(3,37) = 16.382, *p*<0.001) and PHASE (*F*(1,37) = 40.449 *p*<0.001) but no GROUP X PHASE interaction (*F*(3,37) = 0.371, *p*>0.05). As is illustrated in Figure 2, post hoc indicated advanced PD took significantly longer than YAC, OAC, and mild PD to complete advance (*ps*<0.001) and withdrawal (*ps*<0.001).

#### (2) Biomechanical measurement of eye tracking

**Figure 3:**
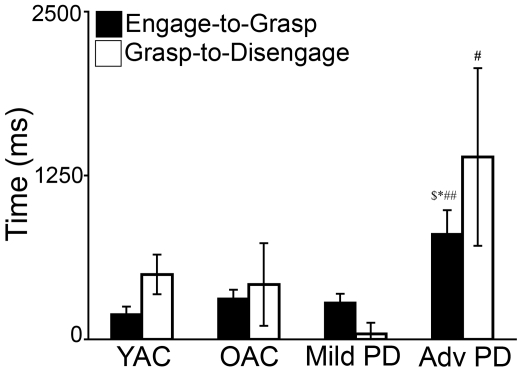
Time (mean and standard error) from engage to move and grasp to disengage for the four experimental groups. ▒ = PD>YAC; * = advanced PD>OAC; # = advanced PD>mild PD, ps<0.0001.

The results of the biomechanical measurements of eye tracking are summarized in Figure 3. A 4×2 ANOVA was performed on movement time using GROUP (YAC, OAC, Mild PD, Advanced PD) and EYE MEASURE (engage to move, grasp to disengage) as independent variables. There was a significant effect of GROUP (*F*(3,37) = 4.616, *p*<0.01). The EYE MEASURE (*F*(1,37) = 0.269, *p*>0.05) and the GROUP X EYE MEASURE (*F*(3,37) = 0.365, *p*>0.05) effects were not significant. As presented in Figure 3, post hoc comparisons indicated advanced PD took longer than YAC, OAC, and mild PD to complete engage to move (*ps*<0.001) and took longer than mild PD to complete grasp to disengage (*p*<0.05).

#### (3) Movement element scoring

**Figure 4:**
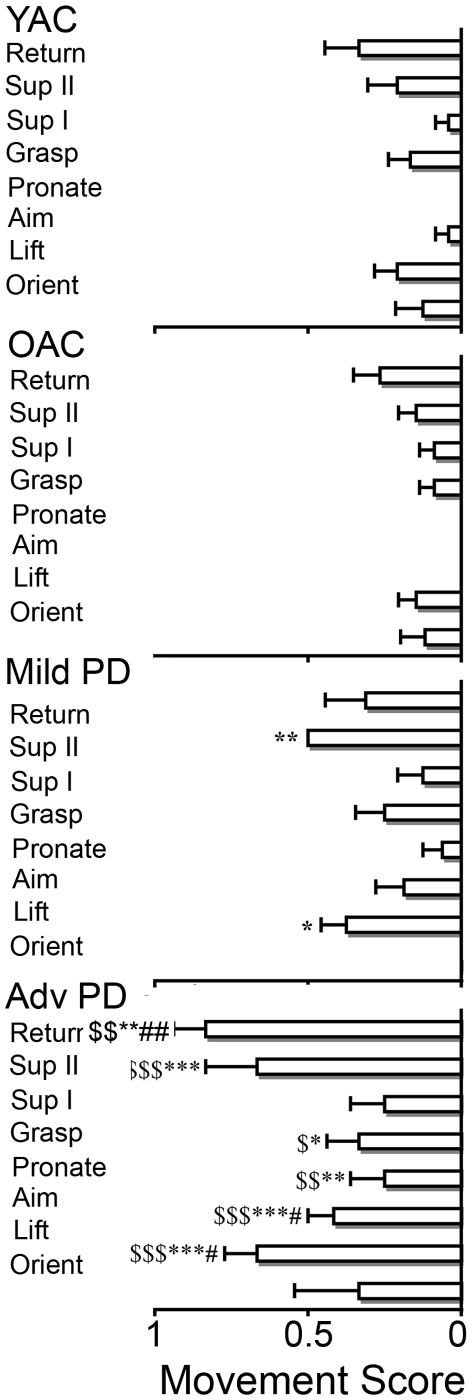
Movement element score (mean and standard error) for the four experimental groups. ▒ = advanced PD>YAC; ▒▒ = advanced PD>OAC; * = advanced PD>mild PD.

The results of the movement element scoring are summarized in Figure 4. A 4×8×2 ANOVA was performed on the movement score using GROUP (YAC, OAC, Mild PD, Advanced PD), MOVEMENT ELEMENT (orient, lift, aim, pronate, grasp, supination I, supination II, return), and CONDITION (vision, occlusion) as independent variables. There was a significant effect for GROUP (*F*(3,70) = 37.098, *p*<0.001), CONDITION (*F*(1,70) = 55.490, *p*<0.001), CONDITION X MOVEMENT ELEMENT (*F*(7,504) = 24.876, *p*<0.001), and GROUP X MOVEMENT ELEMENT (*F*(21,504) = 1.996, *p*<0.01) effects. The GROUP X CONDITION interaction (*F*(3,70) = 1.902, *p*>0.05) was not significant. As shown in Figure 4, post hoc analysis indicated that advanced PD group had higher scores than did all of the other groups on most movement elements, except orient and supination I. The mild PD group had higher element scores than did the OAC and YAC on two measures, lift and supination II.

### Relation between biomechanical measurement of reaching and movement element scoring

**Figure 5:**
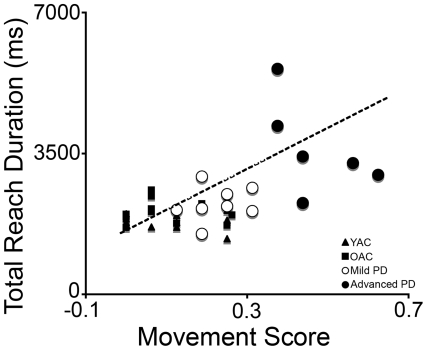
Correlation between movement score and total reach duration. The line represents the regression of the Parkinson's diseased groups combined. Note the close relationship between the two measures.

The results of the relation between the biomechanical measurement of reaching and movement element scoring are summarized in Figure 5. Briefly, the subjects who took the longest to reach had the highest movement element scores. This was confirmed with a Spearman's rho significant correlation of total reach duration and total movement score for all subjects (*rho* = 0.468, *p*<0.01). Correlation between reach time and movement element score for all control subjects (young and old) was not significant (rho = −0.003, *p*>0.05). Correlation between reach time and movement element score for all PD subjects (mild and advanced) was significant (*rho* = 0.643, *p*<0.01).

### Experiment 2: Skilled reaching with visual occlusion

The biomechanical measures of reaching indicated that the advance phase of reaching slowed with visual occlusion for all subjects. The withdrawal phase of skilled reaching was unaffected by visual occlusion, with the exception of advanced PD subjects. They were additionally slowed under visual occlusion. The movement element scoring indicated that visual occlusion impaired control and mild PD subjects relative to reaching with vision. Under visual occlusion they no longer shaped the hand in advance of grasping. Movement element scores were not additionally raised by visual occlusion for the advanced PD subjects, who displayed little hand shaping under either vision or occluded conditions. These main findings were confirmed statistically as is described fully below.

#### (1) Biomechanical measures of reaching

**Figure 6:**
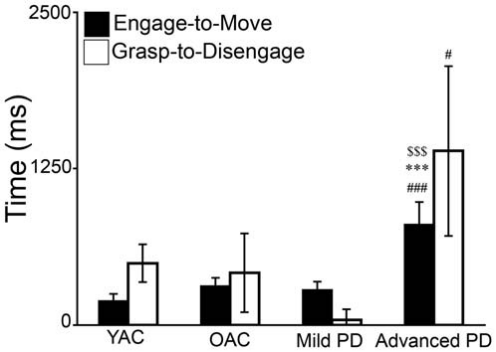
Time to complete advance and withdrawal (mean and standard errors) for the four experimental groups. * = different from visual condition (*<0.05; **<0.01; ***<0.001). Note for the advanced PD group both advance and withdrawal shows a significant change.

The results of the biomechanical measurements of reaching are summarized in Figure 6. A 4×2×2 repeated measures ANOVA was performed on movement time using GROUP (YAC, OAC, Mild PD, Advanced PD) as the between subjects measure and PHASE (advance, withdrawal) and CONDITION (vision, occlusion) as the within subject measure. There was a significant effect of GROUP (*F*(3,70) = 24.525, *p*<0.001), CONDITION (*F*(1,70) = 28.552, *p*<0.001), and CONDITION X PHASE (*F*(1,70) = 28.856, *p*<0.001) effect. The GROUP X PHASE (*F*(3,70) = 2.134, *p*>0.05) interaction was not significant. As presented in Figure 6, post hoc indicated YAC, OAC, mild PD, and advanced PD took longer to complete the advance phase under visual occlusion (*p*<0.001, *p*<0.001, *p*<0.05, *p*<0.05, respectively), while only the advanced PD group took longer to complete the withdrawal phase under visual occlusion (*p*<0.01).

#### (2) Movement element scoring

**Figure 7:**
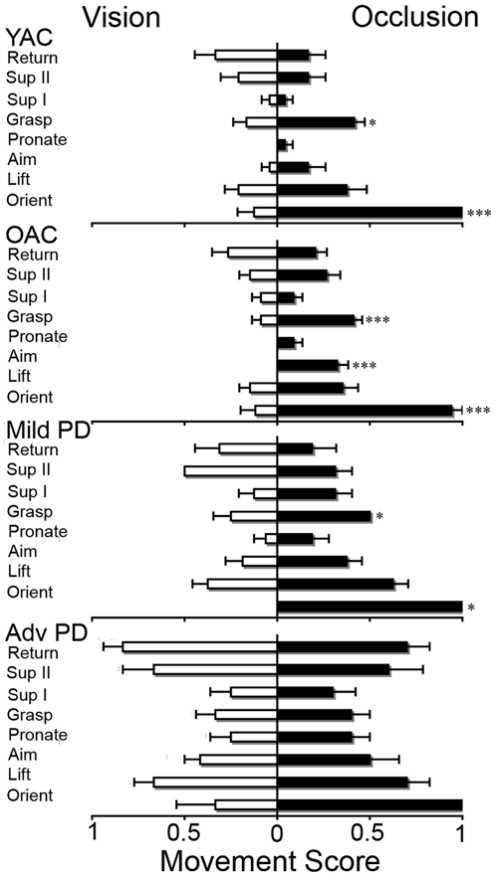
Movement element score (mean and standard error) for vision (left) and occlusion (right). *** = Advanced PD>that other groups; # = Advanced PD>mild PD.

The results of movement element scoring are summarized in Figure 7. Paired t-tests comparing movement elements with vision to those with visual occlusion resulted in a significant effect for occlusion orient and grasp for YAC (*t*(11) = 9.75, *p*<0.001; *t*(11) = 2.16, *p*<0.05, respectively), orient, aim, and grasp for OAC (*t*(16) = 8.64, *p*<0.001; *t*(16) = 5.42, *p*<0.001; *t*(16) = 5.00, *p*<0.001, respectively), orient and lift for mild PD (*t*(7) = 9.80, *p*<0.001; *t*(7) = 2.65, *p*<0.05, respectively) and no difference for advanced PD. As shown in Figure 7, post hoc analysis indicated that advanced PD group was not affected by visual occlusion. The mild PD group had higher scores for orient and lift, age-matched controls had higher scores for orient aim and grasp, and young controls had higher scores for orient and grasp.

### Relation between biomechanical measurement of reaching and movement element scoring under visual occlusion

**Figure 8:**
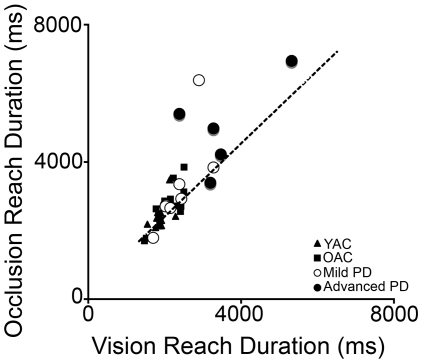
Correlation between total reach duration with vision and total reach duration with occlusion. The line represents the regression of the control groups. Note the disproportionate increase in duration for some PD subjects.

The relation between biomechanical measurements of reaching and movement element scoring under visual occlusion is summarized in Figure 8. Briefly, the subjects who took the longest to reach under visual occlusion also had the highest movement element scores under visual occlusion. This was confirmed with a Spearman's rho significant correlation of total reach duration and total movement score for all subjects (*rho* = 0.863, *p*<0.001). Correlation between reach time and movement element score for all control subjects (young and old) was significant (*rho* = 0.775, *p*<0.001). Correlation between reach time and movement element score for all PD subjects (mild and advanced) was significant (*rho* = 0.797, *p*<0.001).

### Experiment 3: Skilled reaching with preferred musical pieces

The biomechanical measurements of reaching indicated that listening to preferred musical pieces had no effect on the speed of the reaching movement in either the control groups or the PD groups. The biomechanical measurements of eye tracking indicated that listening to preferred musical pieces had no effect on visual guidance of skilled reaching for the control group or the mild PD group. These groups still engaged the target as the reach was initiated and disengaged the target just as it was grasped. Preferred musical pieces did normalize eye movement engagement in the advanced PD group. The advanced PD group decreased the amount of time from visual fixation with the food item both prior to initiating a reach and after the food item was grasped. Nevertheless, movement elements scoring indicated that music did not improve rotary movements of the arm or grasping for PD subjects. These main findings were confirmed statistically as is described fully below.

#### (1) Biomechanical measurement of reaching

**Figure 9:**
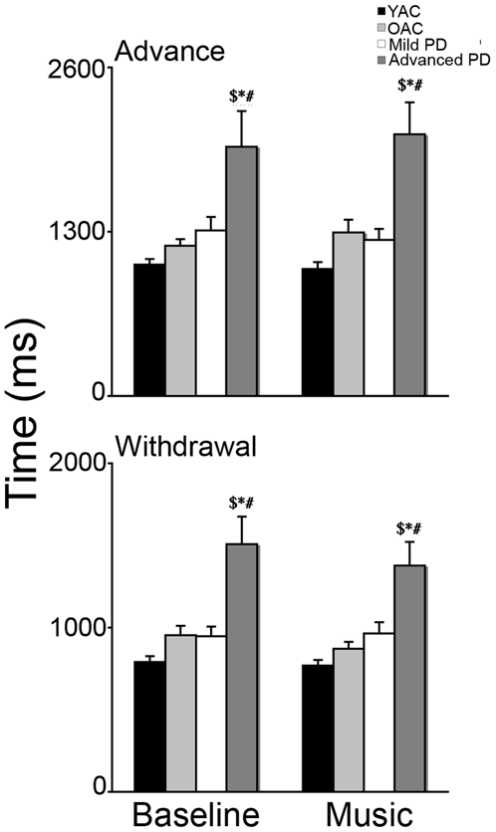
Time to complete advance and withdrawal (mean and standard error) for each group. ▒ = Advanced PD>YAC; * = Advanced PD>OAC; # = advanced PD>mild PD, p<0.001.

The results of the biomechanical measurements of reaching are summarized in Figure 9. A 4×2×2 repeated measures ANOVA was performed on movement time using GROUP (YAC, OAC, Mild PD, Advanced PD) as the between subjects measure and PHASE (advance, withdrawal) and MUSIC (no music, music) as the within subject measure. Analyses revealed a significant effect of GROUP (*F*(3,34) = 15.746, *p*<0.0001), and PHASE (*F*(1,34) = 465.725, *p*<0.0001), but no MUSIC (*F*(1,34) = 0.350, *p*>0.05), GROUP X MUSIC (*F*(3,34) = 0.189, *p*>0.05), or PHASE X MUSIC (*F*(1,34) = 1.683, *p*>0.05) effects. As presented in Figure 9, post hoc comparisons indicated that without accompanying music, advanced PD took longer than YAC, OAC, and mild PD to complete advance (*ps*<0.001), withdrawal (*ps*<0.001), and total reach (*ps*<0.0001). Preferred musical pieces had no effect on these measures as advanced PD continued to take longer than YAC, OAC, and mild PD to complete advance (*ps*<0.001), withdrawal (*ps*<0.001), and the total reach (*ps*<0.001).

#### (2) Biomechanical measurement of eye tracking

**Figure 10:**
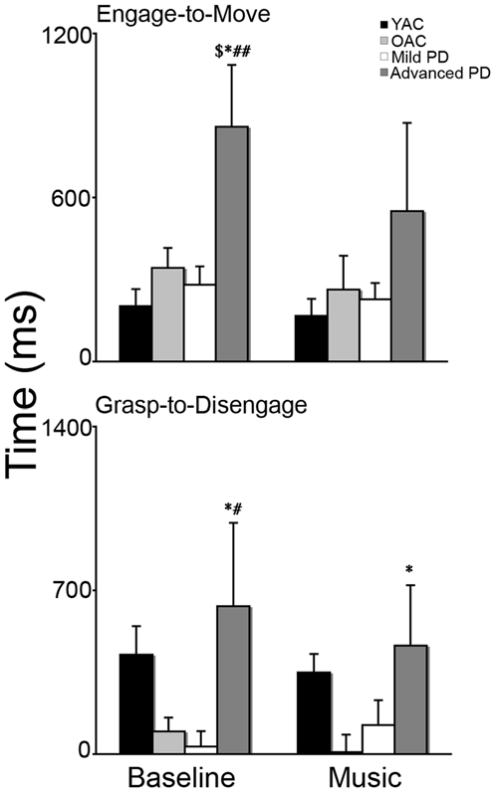
Time to complete engage to move and grasp to disengage (mean and standard error) for the four groups at baseline (left) and with preferred musical pieces (right). ▒ = advanced PD>YAC at p<0.001; * = advanced PD>OAC at p<0.001; # = advanced PD>mild PD at p<0.05.

The results of the biomechanical measurements of eye tracking are summarized in Figure 10. A 4×2×2 repeated ANOVA was performed on the movement time using GROUP (YAC, OAC, Mild PD, Advanced PD) as the between subjects factor and EYE MEASURE (engage to move, grasp to disengage) and MUSIC (no music, music) as the within subjects factors. There was a significant effect of GROUP (*F*(3,34) = 13.259, *p*<0.0001), EYE MEASURE (*F*(2,68) = 279.854, *p*<0.0001), MUSIC (*F*(1,34) = 5.295, *p*<0.05), and GROUP X EYE MEASURE (*F*(6,68) = 9.624, *p*<0.0001) effects, but no GROUP X MUSIC (*F*(3,34) = 1.268, *p*>0.05) effect. As presented in Figure 10, post hoc comparisons without accompanying preferred musical pieces indicated that advanced PD took longer than YAC, OAC, and mild PD to complete engage-to-move (*ps*<0.01), and total engagement duration (*ps*<0.0001). Advanced PD subjects took longer than OAC and mild PD to complete grasp-to-disengage (*ps*<0.05), Post hoc comparisons with accompanying preferred musical pieces indicated that advanced PD subjects took longer than OAC to complete grasp-to-disengage (*p*<0.01), and took longer than YAC, OAC, and mild PD to complete total engagement duration (*ps*<0.0001). There were no significant differences between the groups for engage-to-move (*p*>0.05).

#### (3) Movement element scoring

**Figure 11:**
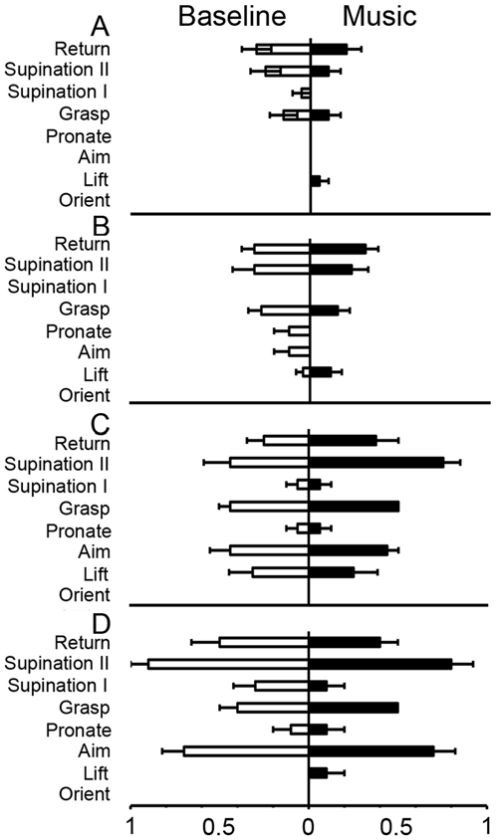
Movement element score (mean and standard error) for reaches completed at baseline (left) and preferred musical pieces (right) for A) YAC; B) OAC; C) mild PD; D) advanced PD. Note that music did not improve movement element scores.

The results of the biomechanical measurements of reaching are summarized in Figure 11. A 4×8×2 repeated ANOVA was performed on the movement score using GROUP (YAC, OAC, Mild PD, Advanced PD) as the between subjects variable and COMPONENT (orient, lift, aim, pronate, grasp, supination I, supination II, return) and MUSIC (no music, music) as the within subjects variable. There was a significant effect of GROUP (*F*(3,34) = 45.365, *p*<0.0001), COMPONENT (*F*(7, 238) = 27.620, *p*<0.0001), GROUP X COMPONENT (*F*(21, 238) = 3.718, *p*<0.0001), and GROUP X MUSIC (*F*(3,34) = 2.945, *p*<0.05) effects, but no MUSIC (*F*(1,34) = 0.707, *p*>0.05), or MUSIC X COMPONENT (*F*(7,238) = 0.479, *p*>0.05) effects. As presented in Figure 11, post hoc for the no music reaches indicated that the PD subjects had higher scores than the controls for lift, aim, supination I, and supination II. Post hoc for reaches with accompanying preferred musical pieces indicated that the PD subjects had higher scores than the controls for aim, pronate, grasp, and supination II.

### Relation between biomechanical measurement of reaching with and without accompanying preferred musical pieces

**Figure 12:**
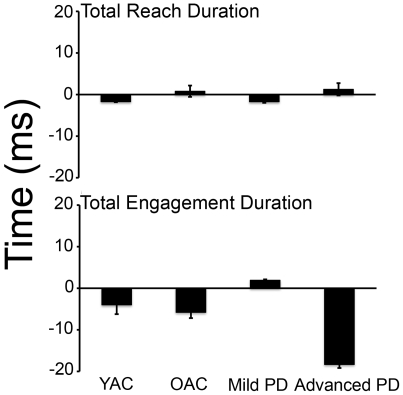
Reach duration (top) and engagement duration (bottom) difference for all experimental groups (no music-music); mean and standard errors. Note that the time to complete total reach duration was not affected by accompanying music, whereas time to complete total engagement duration was decreased for advanced PD subjects.

The relation between biomechanical measurement of reaching with and without accompanying preferred musical pieces is summarized in Figure 12 top. Briefly, the subjects who took the longest to reach without accompanying preferred musical pieces also took the longest to reach with accompanying preferred musical pieces. This was confirmed with a Spearman's rho significant correlation of total reach duration with and without accompanying musical pieces for all subjects (*rho* = 0.909, *p*<0.0001). Correlation between reach time with and without accompanying musical for all control subjects (young and old) was significant (*rho* = 0.845, *p*<0.0001). Correlation between reach time with and without accompanying musical for all PD subjects (mild and advanced) was significant (*rho* = 0.945, *p*<0.0001).

### Relation between biomechanical measurements of eye tracking with and without accompanying musical pieces

The relation between the biomechanical measurements of eye tracking with and without accompanying preferred musical pieces is summarized in Figure 12 bottom. Briefly, subjects who spent the most amount of time visually fixated on the food item without accompanying musical pieces also spent the most time visually fixated on the food item with accompanying musical pieces with the exception of advanced PD subjects who decreased the amount of time spent engaged with the target. This was confirmed with a Spearman's rho significant correlation of biomechanical measurements of eye tracking with and without accompanying musical pieces for all subjects (*rho* = 0.792, *p*<0.0001). Correlation between biomechanical measurements of eye tracking with and without accompanying musical for all control subjects (young and old) was significant (*rho* = 0.498, *p*<0.05). Correlation between movement element scoring with and without accompanying musical for all PD subjects (mild and advanced) was significant (*rho* = 0.954, *p*<0.0001).

### Relation between movement element scoring with and without accompanying preferred musical pieces

Subjects who had the highest movement element scores without accompanying preferred musical pieces also had the highest movement element scores with accompanying preferred musical pieces. This was confirmed with a Spearman's rho significant correlation of movement element scoring with and without accompanying musical pieces for all subjects (*rho* = 0.758, *p*<0.0001). Correlation between movement element scoring with and without accompanying musical for all control subjects (young and old) was not significant (*rho* = 0.360, *p*>0.05). Correlation between movement element scoring with and without accompanying musical for all PD subjects (mild and advanced) was significant (*rho* = 0.537, *p*<0.05).

## Discussion

This study provides the first description of the effect of playing preferred musical pieces on skilled reaching in PD. In order to characterize the effect of music on movement and sensory impairments seen in skilled reaching, subjects were video-recorded as they reached for a small item of food that they then ate while wearing eye-tracking glasses and biomechanical markers. Their reaching movements were additionally rated from frame-by-frame inspection of the video record. The biomechanical measurements of reaching showed that the advanced PD subjects were more impaired than were the control groups, but both mild and advanced PD groups displayed impairments in elements of the reach movement, especially in aiming, rotation of the limb, and grasping. The biomechanical measurements of reaching in the absence of vision showed that all groups were impaired in the advance phase of the reach, but only the advanced PD subjects were additionally impaired in the withdrawal phase of the reach. When presented with preferred musical pieces, there were no changes in either the biomechanical measures of reaching or the movement element scoring of reaching, but eye movements were normalized in the advanced PD group. The results are discussed in relation to a number of possible interpretations of the action of music on skilled reaching behavior and its sensory control.

The strength of the present study is that it adapts three separate measurements of skilled reaching into a single experimental design. Biomechanical measurements of reaching and movement element scoring produce different information with regard to skilled reaching. Biomechanical measurements reveal impairments in speed and smoothness [8] while movement element scoring reveal impairments in movement normality irrespective of speed [23]–[24]. The current experimental design includes an additional approach for examining skilled reaching, the assessment of sensory guidance using eye-tracking and visual occlusion. Both eye tracking and visual occlusion reveal that limb advance is under direct visual control while withdrawal is not [8], [25]. Thus, the methodology used in the present study provides an assessment of skilled reaching in healthy control subjects and in subjects with PD and should be sensitive to any improvements that may occur as a result of therapeutic manipulations.

Consistent with previous research, the biomechanical measurements of reaching indicated that movement slowed with PD [8], [25]. Control and mild PD subjects did not differ in the time to complete the skilled reaching task, whereas the advanced PD subjects displayed increased movement times for both the advance phase and withdrawal phase of the reach [8]. Similarly, movement element scoring indicated that reaching movements were changed with PD. Young and age-matched control groups displayed no movement impairments on the movement element scoring. Consistent with previous research, both mild and advanced PD subjects display impairment in that they use less rotation of the arm as they advance the hand toward the target, they undershot the target as they brought the hand to it, and they tended to use a whole hand grasping movement. These impairments were greater in the more advanced PD subjects [23]–[24], [26], [29].

Control subjects and mild PD subjects displayed impairment in movement element scores in the absence of visual feedback. When visually occluded, there was less rotation in the limb as the hand was advanced, the digits were not preshaped for grasping, and there was a tendency on the part of subjects to use a whole hand grasp. The advanced PD subjects displayed no additional impairment in movement element scoring because their grasps already featured little rotation, hand shaping and independent digit movement in grasping. A surprising additional deficit displayed by the advanced PD group was that the withdrawal phase of their reach also slowed, a change not observed in control or mild PD groups.

The results of the eye-tracking measurements indicated that eye movements changed with PD. Consistent with previous research, control subjects only engaged the food item as the reach was initiated and they disengaged the moment that the food was contacted by the digits [25]. Eye movements were very similar in the mild PD subjects. It is possible that mild PD subjects might display impairments when off medication, but this was not determined because the design of the study was such that all subjects were on medication. Disengagement was usually associated with an eye blink and a visual scan directed to some other part of the test room. Control groups and the mild PD group were not different in this respect. The measurement of eye tracking indicated that advanced PD subjects were different in two ways. First, they engaged the target for an extended period prior to reach initiation. For some trials they simply stared at the food location well before the food was placed there while on other trials they would engage the food and stare at it well before they initiated a reach. Second, they failed to disengage the target as it was grasped and so continued to track the food and hand as the food was transported to the mouth. This impairment is curious in that although visual fixation of the target may stem from the akinesia of PD, it is less clear that the failure to disengage can be similarly explained.

The playing of preferred musical pieces during skilled reaching trials did not affect movement as assessed by the biomechanical measurements or scores on movement elements in any of the groups. That playing preferred musical pieces did not have an effect on the biomechanical measurements of reaching nor movement element scoring is consistent with previous literature showing that music without an embedded rhythmic auditory stimulation does not improve movement execution [15], [30]. Significant improvements in cadence, stride length, and velocity of gait emerged only in the rhythmic auditory stimulation group, whereas the non-embedded music and no music groups did not show any improvement [15]. Similarly, PD performance on a forelimb task of reaching for a pen and bringing it to paper has been reported to show no improvement in movement execution with music with an embedded auditory stimulation [31].

Preferred musical pieces did affect eye movements of the advanced PD subjects. When listening to preferred musical pieces, their eye tracking movements were normalized in that they engaged the target concurrent with reach initiation and disengaged quickly after the target was grasped. Thus, music made the eye movements of the advanced PD subjects very similar to the movements of the control group and the mild PD group. There are at least three interpretations why playing preferred musical pieces had a beneficial effect on eye tracking. First, music may act as a cue to shift visual attention from one locus to another [32]–[34]. Visual cueing improves the reaction time of PD subjects on a task in which subjects save a cartoon character from getting run over [35]. Additionally, if external cueing is reduced following trials with external cueing, PD performance becomes impaired compared to control performance on a button-to-button push task [36]. Second, music may normalize the balance between visual and proprioceptive guidance. Playing preferred musical pieces improves motor initiation in hemi-paretic stroke patients on a task in which subjects were asked to reach out, touch a sensor, and return their arm to the start position [37]. Similarly, PD subjects show an increased force and velocity of initial steps during gait when presented with a cutaneous “go” signal during a step initiation gait task [38]. Third, music may act to activate arousal. Hu and colleagues have suggested that the auditory system is comprised of two pathways. The lemniscal pathway is hypothesized to be responsible for tonotopic processing of auditory information, whereas the nonlemniscal pathway is responsible for other aspects of auditory processing, including the activating effects of audition. The nonlemniscal system is proposed bypass the deficient basal ganglia circuitry responsible for PD and so relieve some symptoms of PD when activated by music [39]–[41].

In conclusion, the results of the present study show that the presentation of preferred musical pieces did not have an effect on either the speed of skilled reaching, nor the movement elements that comprise it. The advanced PD group did, however, display impairment in the visual control of reaching in that they tended to fixate the target both before reach initiation and after grasping and this deficit was ameliorated by music. Because vision is important to many aspects of behavior including adjusting posture, walking, and manipulating objects, it is possible that normalization of visual tracking could improve performance on many tasks. This idea could be investigated in future studies featuring larger groups of PD subjects, measures of performance on different tasks, and the relation between medication, movement, and visual control.
